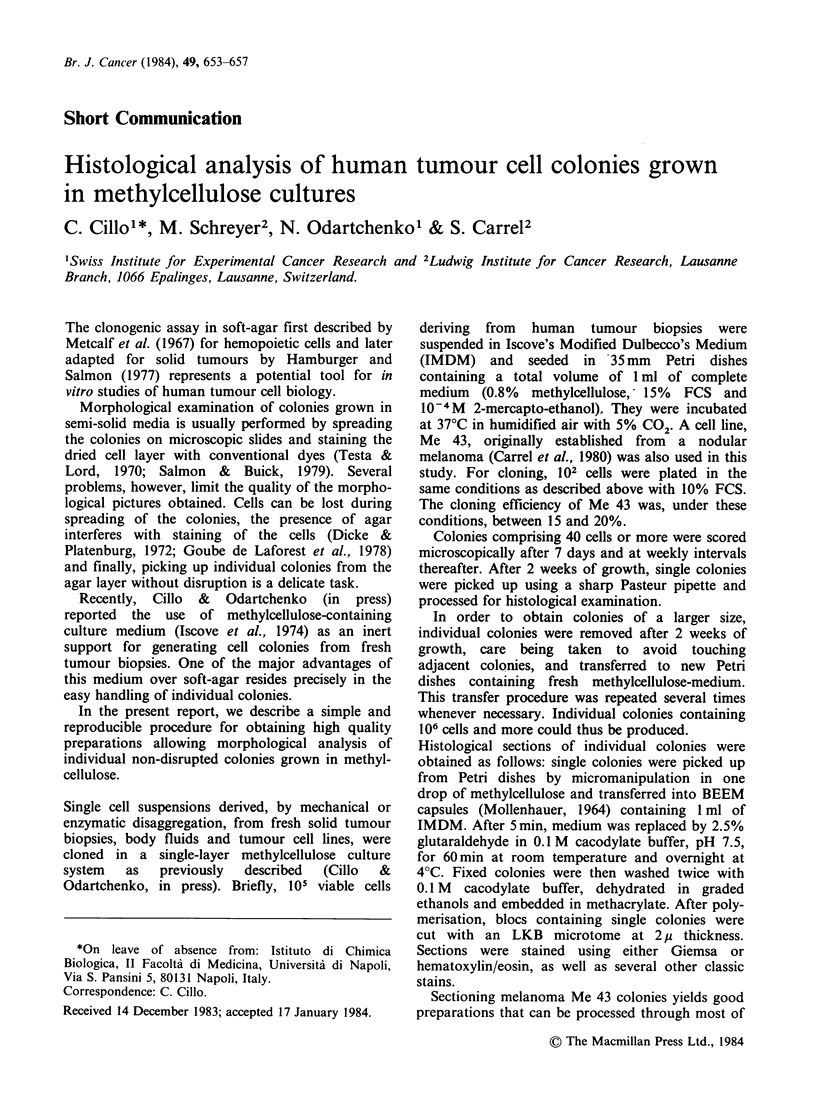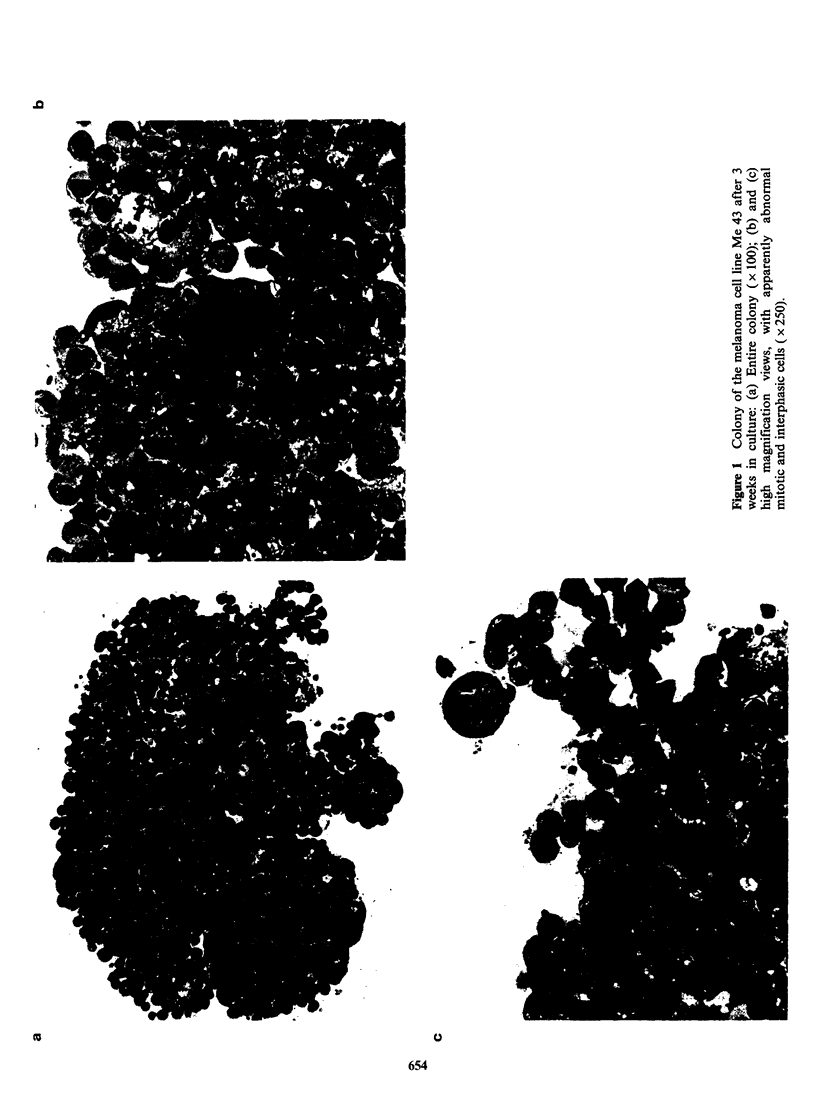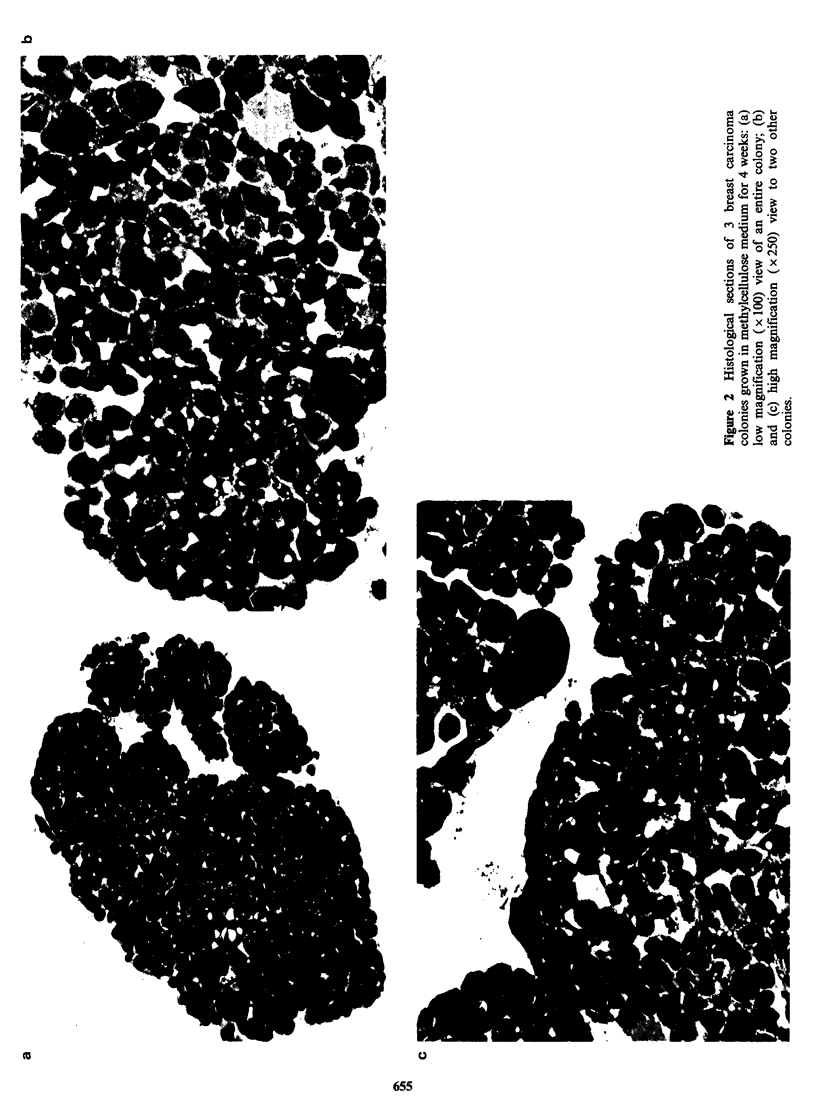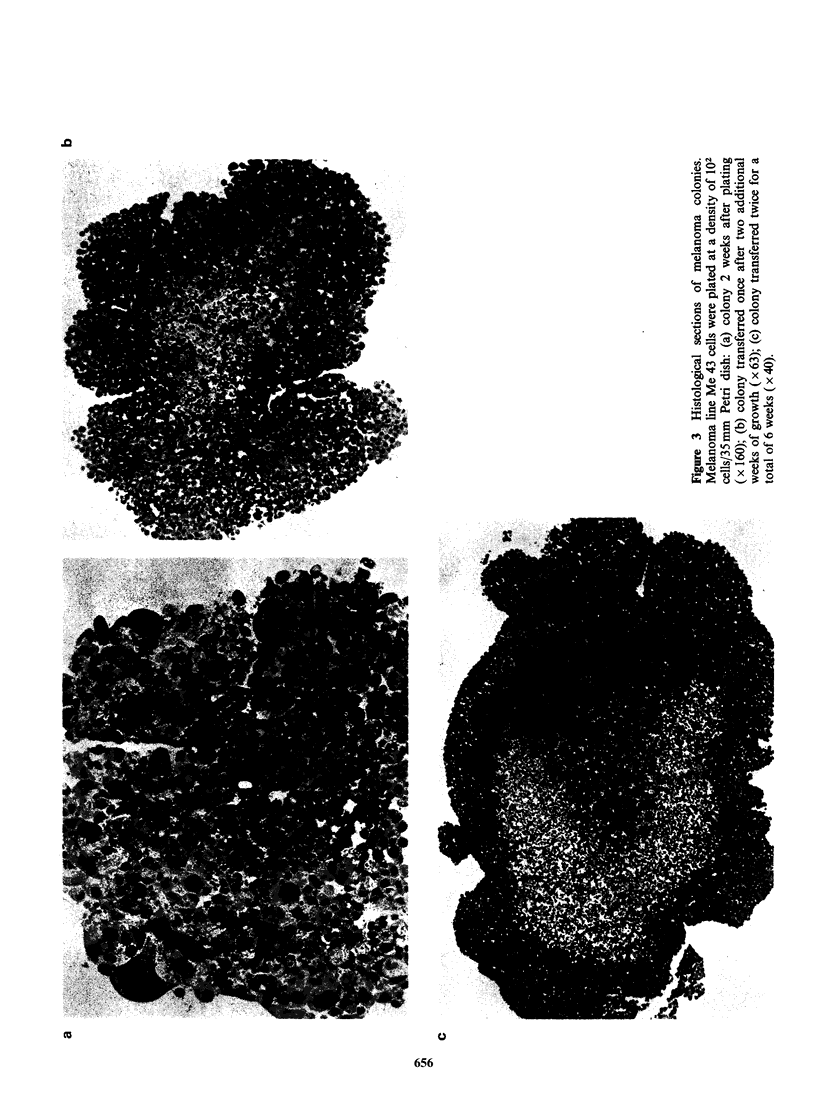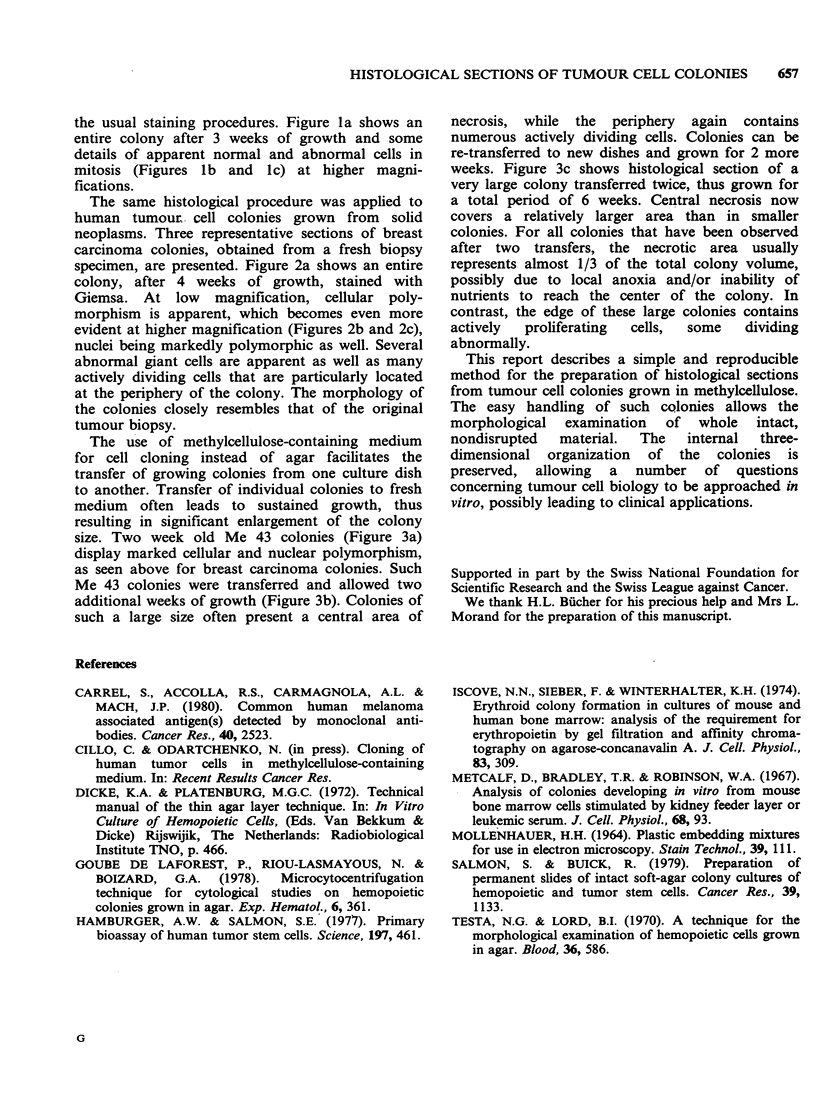# Histological analysis of human tumour cell colonies grown in methylcellulose cultures.

**DOI:** 10.1038/bjc.1984.101

**Published:** 1984-05

**Authors:** C. Cillo, M. Schreyer, N. Odartchenko, S. Carrel

## Abstract

**Images:**


Br. J. Cancer (1984), 49, 653–657

**Short Communication**

### Histological analysis of human tumour cell colonies grown in methylcellulose cultures

C. Cillo[1]*, M. Schreyer[2], N. Odartchenko[1] & S. Carrel[2]

[1]*Swiss Institute for Experimental Cancer Research and* [2]*Ludwig Institute for Cancer Research, Lausanne Branch, 1066 Epalinges, Lausanne, Switzerland.*

The clonogenic assay in soft-agar first described by Metcalf *et al.* (1967) for hemopoietic cells and later adapted for solid tumours by Hamburger and Salmon (1977) represents a potential tool for *in vitro* studies of human tumour cell biology.

Morphological examination of colonies grown in semi-solid media is usually performed by spreading the colonies on microscopic slides and staining the dried cell layer with conventional dyes (Testa & Lord, 1970; Salmon & Buick, 1979). Several problems, however, limit the quality of the morphological pictures obtained. Cells can be lost during spreading of the colonies, the presence of agar interferes with staining of the cells (Dicke & Platenburg, 1972; Goube de Laforest *et al.*, 1978) and finally, picking up individual colonies from the agar layer without disruption is a delicate task.

Recently, Cillo & Odartchenko (in press) reported the use of methylcellulose-containing culture medium (Iscove *et al.*, 1974) as an inert support for generating cell colonies from fresh tumour biopsies. One of the major advantages of this medium over soft-agar resides precisely in the easy handling of individual colonies.

In the present report, we describe a simple and reproducible procedure for obtaining high quality preparations allowing morphological analysis of individual non-disrupted colonies grown in methylcellulose.

Single cell suspensions derived, by mechanical or enzymatic disaggregation, from fresh solid tumour biopsies, body fluids and tumour cell lines, were cloned in a single-layer methylcellulose culture system as previously described (Cillo & Odartchenko, in press). Briefly, $10^5$ viable cells deriving from human tumour biopsies were suspended in Iscove's Modified Dulbecco's Medium (IMDM) and seeded in 35 mm Petri dishes containing a total volume of 1 ml of complete medium (0.8% methylcellulose, 15% FCS and $10^{-4}$ M 2-mercapto-ethanol). They were incubated at 37°C in humidified air with 5% $CO_2$. A cell line, Me 43, originally established from a nodular melanoma (Carrel *et al.*, 1980) was also used in this study. For cloning, $10^2$ cells were plated in the same conditions as described above with 10% FCS. The cloning efficiency of Me 43 was, under these conditions, between 15 and 20%.

Colonies comprising 40 cells or more were scored microscopically after 7 days and at weekly intervals thereafter. After 2 weeks of growth, single colonies were picked up using a sharp Pasteur pipette and processed for histological examination.

In order to obtain colonies of a larger size, individual colonies were removed after 2 weeks of growth, care being taken to avoid touching adjacent colonies, and transferred to new Petri dishes containing fresh methylcellulose-medium. This transfer procedure was repeated several times whenever necessary. Individual colonies containing $10^6$ cells and more could thus be produced.

Histological sections of individual colonies were obtained as follows: single colonies were picked up from Petri dishes by micromanipulation in one drop of methylcellulose and transferred into BEEM capsules (Mollenhauer, 1964) containing 1 ml of IMDM. After 5 min, medium was replaced by 2.5% glutaraldehyde in 0.1 M cacodylate buffer, pH 7.5, for 60 min at room temperature and overnight at 4°C. Fixed colonies were then washed twice with 0.1 M cacodylate buffer, dehydrated in graded ethanols and embedded in methacrylate. After polymerisation, blocs containing single colonies were cut with an LKB microtome at 2 $\mu$ thickness. Sections were stained using either Giemsa or hematoxylin/eosin, as well as several other classic stains.

Sectioning melanoma Me 43 colonies yields good preparations that can be processed through most of

*On leave of absence from: Istituto di Chimica Biologica, II Facoltà di Medicina, Università di Napoli, Via S. Pansini 5, 80131 Napoli, Italy.
Correspondence: C. Cillo.

Received 14 December 1983; accepted 17 January 1984.

© The Macmillan Press Ltd., 1984

the usual staining procedures. Figure 1a shows an entire colony after 3 weeks of growth and some details of apparent normal and abnormal cells in mitosis (Figures 1b and 1c) at higher magnifications.

The same histological procedure was applied to human tumour cell colonies grown from solid neoplasms. Three representative sections of breast carcinoma colonies, obtained from a fresh biopsy specimen, are presented. Figure 2a shows an entire colony, after 4 weeks of growth, stained with Giemsa. At low magnification, cellular polymorphism is apparent, which becomes even more evident at higher magnification (Figures 2b and 2c), nuclei being markedly polymorphic as well. Several abnormal giant cells are apparent as well as many actively dividing cells that are particularly located at the periphery of the colony. The morphology of the colonies closely resembles that of the original tumour biopsy.

The use of methylcellulose-containing medium for cell cloning instead of agar facilitates the transfer of growing colonies from one culture dish to another. Transfer of individual colonies to fresh medium often leads to sustained growth, thus resulting in significant enlargement of the colony size. Two week old Me 43 colonies (Figure 3a) display marked cellular and nuclear polymorphism, as seen above for breast carcinoma colonies. Such Me 43 colonies were transferred and allowed two additional weeks of growth (Figure 3b). Colonies of such a large size often present a central area of necrosis, while the periphery again contains numerous actively dividing cells. Colonies can be re-transferred to new dishes and grown for 2 more weeks. Figure 3c shows histological section of a very large colony transferred twice, thus grown for a total period of 6 weeks. Central necrosis now covers a relatively larger area than in smaller colonies. For all colonies that have been observed after two transfers, the necrotic area usually represents almost 1/3 of the total colony volume, possibly due to local anoxia and/or inability of nutrients to reach the center of the colony. In contrast, the edge of these large colonies contains actively proliferating cells, some dividing abnormally.

This report describes a simple and reproducible method for the preparation of histological sections from tumour cell colonies grown in methylcellulose. The easy handling of such colonies allows the morphological examination of whole intact, nondisrupted material. The internal three-dimensional organization of the colonies is preserved, allowing a number of questions concerning tumour cell biology to be approached *in vitro*, possibly leading to clinical applications.

Supported in part by the Swiss National Foundation for Scientific Research and the Swiss League against Cancer.

We thank H.L. Bücher for his precious help and Mrs L. Morand for the preparation of this manuscript.

**References**

CARREL, S., ACCOLLA, R.S., CARMAGNOLA, A.L. & MACH, J.P. (1980). Common human melanoma associated antigen(s) detected by monoclonal antibodies. *Cancer Res.*, **40**, 2523.

CILLO, C. & ODARTCHENKO, N. (in press). Cloning of human tumor cells in methylcellulose-containing medium. In: *Recent Results Cancer Res.*

DICKE, K.A. & PLATENBURG, M.G.C. (1972). Technical manual of the thin agar layer technique. In: *In Vitro Culture of Hemopoietic Cells,* (Eds. Van Bekkum & Dicke) Rijswijik, The Netherlands: Radiobiological Institute TNO, p. 466.

GOUBE DE LAFOREST, P., RIOU-LASMAYOUS, N. & BOIZARD, G.A. (1978). Microcytocentrifugation technique for cytological studies on hemopoietic colonies grown in agar. *Exp. Hematol.*, **6**, 361.

HAMBURGER, A.W. & SALMON, S.E. (1977). Primary bioassay of human tumor stem cells. *Science*, **197**, 461.

ISCOVE, N.N., SIEBER, F. & WINTERHALTER, K.H. (1974). Erythroid colony formation in cultures of mouse and human bone marrow: analysis of the requirement for erythropoietin by gel filtration and affinity chromatography on agarose-concanavalin A. *J. Cell. Physiol.*, **83**, 309.

METCALF, D., BRADLEY, T.R. & ROBINSON, W.A. (1967). Analysis of colonies developing *in vitro* from mouse bone marrow cells stimulated by kidney feeder layer or leukemic serum. *J. Cell. Physiol.*, **68**, 93.

MOLLENHAUER, H.H. (1964). Plastic embedding mixtures for use in electron microscopy. *Stain Technol.*, **39**, 111.

SALMON, S. & BUICK, R. (1979). Preparation of permanent slides of intact soft-agar colony cultures of hemopoietic and tumor stem cells. *Cancer Res.*, **39**, 1133.

TESTA, N.G. & LORD, B.I. (1970). A technique for the morphological examination of hemopoietic cells grown in agar. *Blood*, **36**, 586.